# Estimating illegal fishing from enforcement officers

**DOI:** 10.1038/s41598-020-69311-5

**Published:** 2020-07-27

**Authors:** C. Josh Donlan, Chris Wilcox, Gloria M. Luque, Stefan Gelcich

**Affiliations:** 1 Advanced Conservation Strategies, Midway, UT 84049 USA; 2 Cornell Lab of Ornithology, Cornell University, Ithaca, NY 14850 USA; 3 CSIRO Oceans and Atmosphere, Castray Esplanade, Hobart, TAS Australia; 4 Center of Applied Ecology and Sustainability (CAPES) & Center for the Study of Multiple-Drivers on Marine Socio-Ecological Systems (MUSELS), Pontificia Universidad Católica de Chile, Santiago, Chile

**Keywords:** Environmental impact, Conservation biology, Marine biology

## Abstract

While illegal, unreported, and unregulated (IUU) fishing is a premier issue facing ocean sustainability, characterizing it is challenging due to its clandestine nature. Current approaches can be resource intensive and sometimes controversial. Using Chile as an example, we present a structured process leveraging existing capacity, fisheries officers, that provides a monitoring tool to produce transparent and stand-alone estimates on the level, structure, and characteristics of illegal fishing. We provide a national illegal fishing baseline for Chile, estimating illegal activity for 20 fisheries, representing ~ 70% of annual national landings. For four fisheries, we also estimate the relative importance of illegal activities across sectors, stakeholders, and infrastructure. While providing new information, our results also confirm previous evidence on the general patterns of illegality. Our approach provides an opportunity for government agencies to formalize their institutional knowledge, while accounting for potential biases and reducing fragmentation of knowledge that can prevent effective enforcement. Estimating illegal activity directly from fisheries enforcement officers is complementary to existing approaches, providing a cost-effective, rapid, and rigorous method to measure, monitor, and inform solutions to reduce IUU fishing.

## Introduction

Illegal, unreported, and unregulated (IUU) fishing is one of the most pressing issues impacting fisheries management and the conservation of marine biodiversity^1,2^. In some cases, it contributes directly to species endangerment: examples include abalone (*Haliotis midae*) in South Africa and multiple species of sturgeon (Acipenseridae) throuhgout their range^3,4^. More broadly, IUU activities directly inhibit the recovery and sustainable management of fisheries stocks and precipitate indirect impacts on biodiversity through unsustainable fishing practices^5,6^. They are also often connected to labor abuses, as well as the loss of billions of dollars in economic benefits^7,8^. Thus, reducing IUU fishing is a national priority for many countries and doing so is considered to be a high-benefit means of improving the state of many fisheries^9^.

While studies that attempt to do so have increased over the past decade, e.g.,^7,10–13^, characterizing IUU fishing is challenging due to its clandestine nature and scarcity of data. Illegal catch is rarely, if ever, known. Rather, IUU estimation is commonly tackled using two approaches: statistical accounting and evidence-based estimation^6^. In general, the former uses various catch and trade data in an attempt to estimate unreported catch. This approach is often challenging due to the quality of trade data, market complexity, lack of conversion factors, and geographical coverage of data^6^. Estimation approaches also have issues. For example, the accuracy of estimates is sometimes low or unknown, and some approaches include many assumptions and lack transparency^6,11^. Surveillance data, stock assessments based on survey data, sensitive survey methodologies, and expert opinion have also been used to inform illegal fishing^6,14,15^. Researchers have also developed methods to combine multiple datasets. For example, the *anchor point and influence* method generates IUU estimates across space and time by combining multiple datasets and data types, along with Monte Carlo simulations^7,15,16^. In some cases^13,17,18^, this methodology has been criticized and its estimates challenged due to lack of transparency and potential biases^19–22^. Regardless, this approach, along with others, are time- and resource-intensive and its replicability across space and time can be challenging.^11^ For many stakeholders (e.g., fisheries management agencies), there is desire to complement existing IUU assessments with regular and repeatable information on illegal fishing that is attainable with limited time and resources.

There is substantial informal information within fisheries communities that can provide a basis for evaluating illegal fishing. For example, sensitive survey techniques have been used to estimate IUU activity directly from fishers^15,23,24^. Fisheries managers, enforcement officers, and other government officials also hold a wealth of informal information, which presents an opportunity to use elicitation protocols to formalize that knowledge in a structured manner^25^. Expert elicitation techniques have been increasingly used in the conservation and sustainability sciences to inform decisions on issues with insufficient data^26–28^. The approach is particularly useful in estimating risks and prioritizing interventions^29^. While fishers' perceptions and expert opinion has been integrated with fisheries data to estimate illegal catch^12,16,18,30^, a formal elicitation process using fisheries enforcement officials, to our knowledge, has not been used to derive stand-alone estimates of illegal activity.

We present the results of an expert elicitation conducted with enforcement officials in Chile's national fisheries agency (Servicio Nacional de Pesca y Acuicultura, SERNAPESCA). Illegal fishing activity is considered high in Chile and is a priority of the national government^31,32^. We conducted a survey focused on gathering synoptic information on fishery-specific illegal activity and the characteristics of those activities, along with the relative importance of different stakeholders and infrastructure across the seafood supply chain. In this paper, we present three main results that together provide a national baseline on illegal fishing for Chile. First, we estimate relative illegal activity for 20 fisheries, which accounts for approximately 80% of annual landings^33^. Our estimates control for variation among respondents and their experience with each fishery. Second, we create illegal profiles for four important Chilean fisheries: two pelagic fish species, a benthic invertebrate, and kelp. For these species, we estimate the relative importance of (1) the types of illegal activity segmented by the small-scale and industrial sectors, (2) the different stakeholders across the supply chain (e.g., wholesaler), and (3) the different infrastructure involved in illegal activity (e.g., restaurants). In doing so, we also control for variation among respondents and their experience with each fishery. Third, we use a multivariate statistical analysis to explore similarities and differences in the illegal landscape for the four focal fisheries. Our primary goal is to demonstrate that a structured process using existing capacity, fisheries enforcement officers, can provide a rapid, cost-effective, and complementary tool to produce transparent estimates on the level, structure, and characteristics of illegal fishing.

## Methods

### Experimental design

Following five focus groups with SERNAPESCA's head of enforcement and other personnel, we designed and implemented an online survey that targeted fisheries enforcement officers who are responsible for monitoring IUU activities in Chile. The survey was structured to capture expert knowledge on various aspects of illegal activities, as well as the relative experience of the officers. The survey defined illegal fishing as *a fishing activity carried out in national jurisdiction waters by national or international boats that is in violation of the national fishing law, conducted without a legal permit, or activities that involve unreported or misreported captures to the authorities. *The Director of SERNAPESCA delivered the survey via email to all SERNAPESCA enforcement officers. The list of officers was constructed by the Director (n = 86). The survey was anonymous in that the officers were not asked to report their name nor any information that could be used for identification (e.g., email). Answers to questions were not mandatory; that is, respondents could opt-out of answering particular questions and continue with the survey. The survey was available online for ten weeks, over which five reminder emails were sent to officers requesting them to complete the survey.

The survey, in Spanish, consisted of two sections. First, we asked respondents to rank the magnitude of illegal activity for twenty fisheries on a nominal scale (1–5), along with their relative experience with each fishery (nominal scale, 1–5). The twenty fisheries were selected a priori based on our focus groups and known information about illegal activity. All fisheries were single species, with the exception of four that included multiple species: skates (2 species, *Zearaja chilensis* and *Bathyraja macloviana*), kelp (4 species: *Lessonia spicate, L. berteroana, L. traberculata, Macrocystis pyrifera*), red algae (3 species: *Sarcothalia crispate, Gigartina skottsbergii, Mazzaella laminarioides*), and crabs (10 species excluding southern king crab: *Cancer edwardsi, C. porter, C. setosus, C. coronatus, Homalaspis plana, Ovalipes trimaculatus, Taliepus dentatus, T. marginatus, Mursia gaudichaudi, Hemigrapsus crenulatus*). In the second part of the survey, we asked respondents additional questions for four focal fisheries: South Pacific hake (*Merluccius gayi gayi*), southern hake (*M. australis*), loco or Chilean abalone (*Concholepas concholepas*), and kelp. For each fishery, we asked respondents to score on a nominal scale (1–5),

- The frequency of six specific illegal activities in the industrial sector: size, gear, season, quota, transshipment, and port.
- The frequency of six specific illegal activities in the small-scale sector: size, gear, season, quota, transshipment, and port.
- The participation of illegal activity for six different stakeholders along the supply chain: fisher, purchaser, processor, wholesaler, exporter, and restaurateur.
- The utilization of seven infrastructure types in illegal activities: fishing boats, refrigeration trucks, processing plants, markets, transshipment boats, export vehicles, and restaurants.

This study was approved by the Advanced Conservation Strategies and Pontificia Universidad Católica ethics institutional review boards and followed guidelines established by their ethics committees, which complies with national and international standards. The surveys included a written informed consent approved by all interviewees, which acknowledged research objectives and established that the survey was anonymous and that interviewees were free to choose to not answer questions. While all species have common names in Chile (which were used in the survey), we use Fishbase and Sealifebase as the taxonomic authority and for the common names reported here to facilitate comparisions^34,35^.

### Statistical analysis

For both sections of the survey, we used a Bayesian cumulative multinomial logit model to predict illegal estimates. First, we fitted a model for illegal estimates for each of the twenty fisheries jointly. Second, we fitted models for illegal estimates for various aspects of the four focal fisheries (i.e., activities, stakeholders, and infrastructure) in a single analysis for each aspect. In both models, we included a random intercept term for respondent, along with a fixed effect for fishery. We evaluated the role of experience, as self-reported by the respondents, by comparing the difference between the illegal score by a respondent for a fishery and the model prediction for that fishery across respondents. If higher levels of expertise increased or decreased the value of a respondent's scoring, there would be a relationship between the size of the differences and the level of experience reported for a fishery. Experience may also affect the difference in mean responses (i.e., bias), potentially due to more personal experience over a longer period of time, which would lead to a correlation between expertise and mean illegality scores. Depending on the patterns observed in the data, there are several ways to control for a respondent's experience in illegality estimates. In our case, we used experience scores as a covariate in the model.

For the twenty fisheries, we used the following model,

1 $$Pr\left\{{S}_{ij}=k\right\}=\phi \left({\tau }_{k}-\left({\varvec{\beta}}{{\varvec{x}}}_{{\varvec{i}}}+{{\varvec{z}}}_{{\varvec{j}}}{{\varvec{V}}}_{{\varvec{i}}}\right)\right)-\phi \left({\tau }_{k-1}-\left({\varvec{\beta}}{{\varvec{x}}}_{{\varvec{i}}}+{{\varvec{z}}}_{{\varvec{j}}}{{\varvec{V}}}_{{\varvec{j}}}\right)\right)$$

in which the probability that the score for the level of illegal landings ${S}_{ij}$ for the *ith* species by the *jth* respondent is equal to category k, can be represented as a latent continuous variable which is divided into K categories, by K − 1 thresholds at ${\tau }_{k}$. This latent continuous variable is represented by the cumulative normal distribution, $\phi$. For a given observation, the regression equation is composed of coefficients multiplied times predictor variables ${\varvec{\beta}}{{\varvec{x}}}_{{\varvec{i}}}$ plus a design matrix for the random effect, multiplied times the error term for the jth respondent, ${{\varvec{z}}}_{{\varvec{j}}}{{\varvec{V}}}_{{\varvec{i}}}$ . The probability of that observation falling in category k, $Pr\left\{{S}_{ij}=k\right\}$, is thus the probability of it being in a category equal to or smaller than k, $\phi \left({\tau }_{k}-\left({\varvec{\beta}}{{\varvec{x}}}_{{\varvec{i}}}+{{\varvec{z}}}_{{\varvec{j}}}{{\varvec{V}}}_{{\varvec{i}}}\right)\right)$, less the probability of the observation being in a category smaller than k, $\phi \left({\tau }_{k-1}-\left({\varvec{\beta}}{{\varvec{x}}}_{{\varvec{i}}}+{{\varvec{z}}}_{{\varvec{j}}}{{\varvec{V}}}_{{\varvec{j}}}\right)\right)$. Implemented in the R statistical language, using the *brms* package^36^, the call to fit this model looks like the following:

$${\text{Score}}\; \, \sim \;{\text{Species}} + {\text{Experience }} + \left( {{1}|{\text{Respondent}}} \right),\;{\text{ data}} = {\text{SurveyData}},\;{\text{family}} = {\text{cumulative}}),$$

where Score is ${S}_{ij}$ in (1) above, the fixed effects, ${\varvec{\beta}}{{\varvec{x}}}_{{\varvec{i}}}$ are the experience of the respondent and the species that was scored, and (1|Respondent) denotes a random intercept model, where each has a different intercept term, drawn from a shared error distribution. For more information on the application of this model to ordinal response data, see Burkner and Vuorre^37^.

For the estimates for the various aspects of the four focal fisheries, we used the following model,

$${\text{Response}}\; \sim \;{\text{Species}} + {\text{Experience}} + \left( {{1}|{\text{Respondent}}} \right),\;{\text{data}} = {\text{SurveyData}},\;{\text{family}} = {\text{cumulative}}),$$

which is structured as per (1) above, but with the responses to the various focal species questions (i.e., activities per sector, stakeholders, and infrastructure) substituted for the species scores as in (1).

We compared both models with simpler models, including a single-term null model using *leave-one-out cross-validation*. We did so in the R statistical language using the *loo* packages^36,38,39^. Prior distributions for all regression terms were improper flat priors over the real numbers, the default in the *brms* package for population parameters. The priors on the intercept and the random effects were student t_3,0,10_ distributions, as per the default for uninformative priors in the *brms* package.

We carried out a Principal Components Analysis (PCA) with the four focal fisheries as categorical variables and the illegal activity, stakeholder, and infrastructure estimates from the Bayesian cumulative multinomial logit model. For each fishery, we used 10,000 estimates from the model, along with a qualitative variable that represented the different factors (e.g., restaurateur). The latter has no influence on the principal components of the analysis but helps to interpret the dimensions of variability. Principal Components Analysis is especially powerful as an approach to visualize patterns, such as clusters, clines, and outliers in a dataset^40^. In our case, we sought to visualize whether there were common illegal factors with similar set of scores and whether there was any association between high or low scores of illegal factors and the focal fisheries. We used the FactoMineR package in the R statistical language^41^.

## Results

### Response rate

The response rate of the online survey was between 56 and 75%. Sixty-five officers started the online survey, while 48 completed it in its entirety. Because answers to questions were not mandatory, sample size varied across questions. For fisheries-specific scores, the median sample size was 48 (range 45–53). For the scores related to the illegal profiles for the four focal fisheries, the median sample size was 39 (range 30–46).

### Fishery estimates

**Figure 1:**
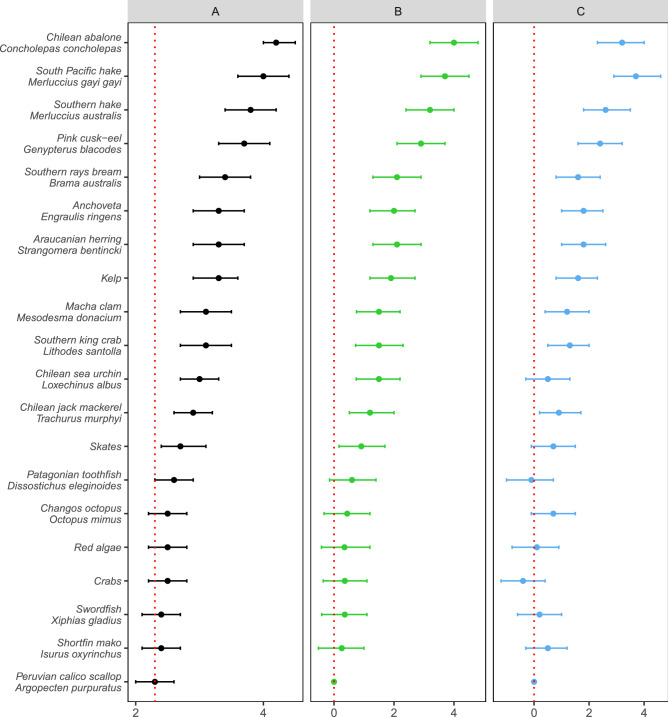
Illegal fishing estimates for 20 Chilean fisheries. (**A**) Mean raw scores (nominal, 1–5) as reported by fishery enforcement officers. (**B**) Predicted estimates from a mixed model with respondent as a random factor. (**C**) Predicted estimates from a mixed model with respondent as a random factor, while also controlling for self-reported experience for each fishery. For (**B**) and (**C**), Peruvian calico scallop is the reference fishery, which also has the lowest mean raw IUU score. Error bars are 95% confidence intervals.

There was variation in the reported level of overall illegal activity across the 20 fisheries. Chilean abalone, followed by two species of hake, had the highest average illegal scores, while the Peruvian calico scallop (*Agropecten purpuratus*) had the lowest (Fig. 1a). The mixed model with fisheries as a fixed effect and respondent as a random effect performed better than the null model (Table S1). The estimates from the model were similar to the raw scores. Thirteen of the twenty fisheries had higher estimates compared with Peruvian calico scallop, which was the reference fishery in the model and had the lowest raw score (Fig. 1b). Because fishery-specific estimates are represented as a fixed effect factor in the model, their estimates can be compared directly. Fisheries with the highest predicted estimates, while controlling for respondent, were Chilean abalone and south Pacific hake, followed by southern hake and red cusk-eel (*Genypterus chilensis*).

There was a negative relationship between respondent experience and his or her score for each fishery. This effect was statistically significant based on a single term linear regression on the residuals from the mixed effects model (Table S2). Thus, we incorporated experience scores as a predictor in the model, which significantly improved model performance (Tables S2, S3). The best approach for incorporating respondent experience was to transform the scores to a linear numerical value, as there was no predictive advantage in treating experience as a categorical or ordinal factor (Table S3). This is likely due to the relatively even spacing of the experience categories, which makes a linear approximation relatively accurate and requires less parameters. After accounting for respondent experience, two of the thirteen predicted IUU estimates were no longer significantly different from the reference fishery: Chilean sea urchin (*Loxechinus albus*) and skates (Fig. 1c). This result suggests that their marginal effects were in part due to the difference in experience among respondents. One fishery, southern hake, had a higher predicted estimate after respondent experience was included in the model.

### Fishery profiles

**Figure 2:**
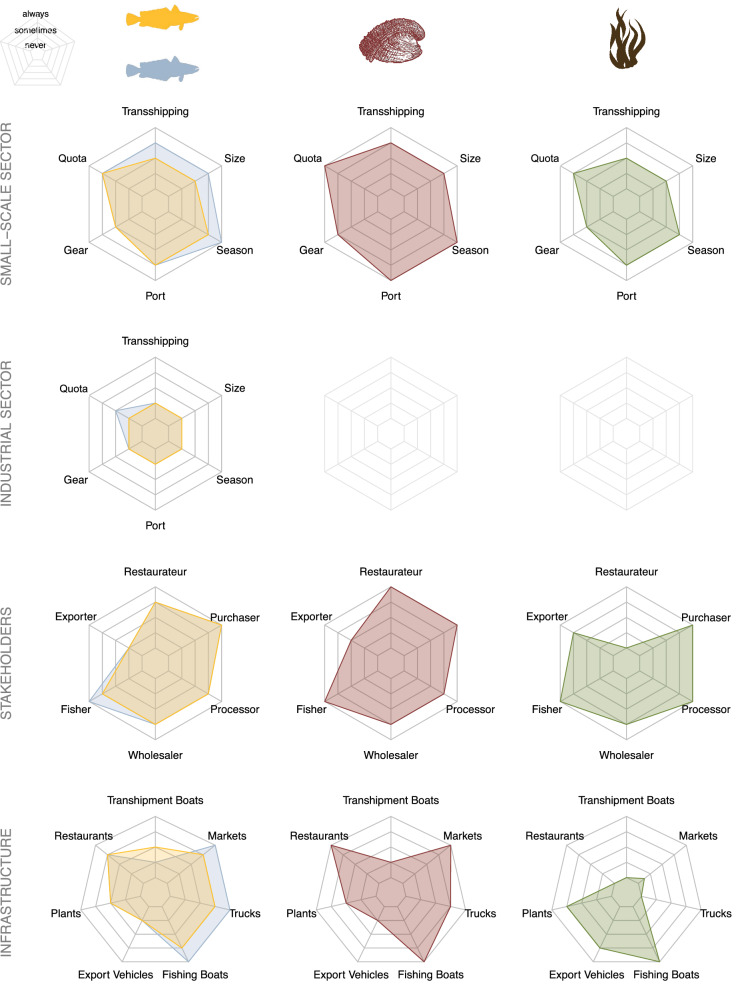
Fishery profiles of four Chilean fisheries: south Pacific hake (blue), southern hake (orange), Chilean abalone (red), and kelp (green). Predicted median estimates of the level of illegality in industrial sector activity, small-scale sector activity, stakeholders, and infrastructure. Industrial fishing does not exist for Chilean abalone and kelp. Predicted medians are from a Bayesian cumulative multinomial logit model for each of the four focal fisheries. The entire posterior distributions of the model results are shown in Figs. S2–S5.

We elicited additional information from respondents on four fisheries that are of particular interest in Chile with respect to illegal activity. For all four fisheries, > 60% of enforcement officers reported that the percentage of total landings that are illegal is greater than 20%, with the most common score being 20–50% for all four fisheries (Fig. S1). Following the model performance from the estimates for the overall illegal activity, we adopted the same model to estimate specific illegal activities, infrastructure, and stakeholders for the focal fisheries: a mixed model with respondent as a random factor and controlling for self-reported experience. The distributions of respondents' experience across the four fisheries were similar. Thus, we standardized activity, infrastructure, and stakeholder scores with a model that included the median experience score (i.e., 4) across the four fisheries. Doing so allows for the comparison of scores across fisheries and different estimate types (e.g., infrastructure versus stakeholders). While industrial fisheries do not exist for Chilean abalone and kelp, estimates for small-scale sector where higher for both species of hake across all illegal activities compared to the industrial sector (Fig. 2). Within the small-scale sector, south Pacific hake had higher estimates for some illegal activities compared to southern hake (i.e., season, size, and transshipping). Illegal activities connected to quota, port, and season had high estimates for Chilean abalone, as well as for kelp (Fig. 2). With respect to stakeholders and infrastructure, there were also differences across the four fisheries. Fishers had high estimates across all fisheries, as did buyers. Restaurateurs had high estimates for Chilean abalone, while processors had high estimates for kelp (Fig. 2). Similarly, fishing boats, markets, and restaurants had high estimates for Chilean abalone, while fishing boats, processing plants, and export vehicles had high estimates for kelp. Fishing boats, trucks, and markets estimates were higher for south Pacific hake compared to southern hake (Fig. 2).

### Principal component analysis

**Figure 3:**
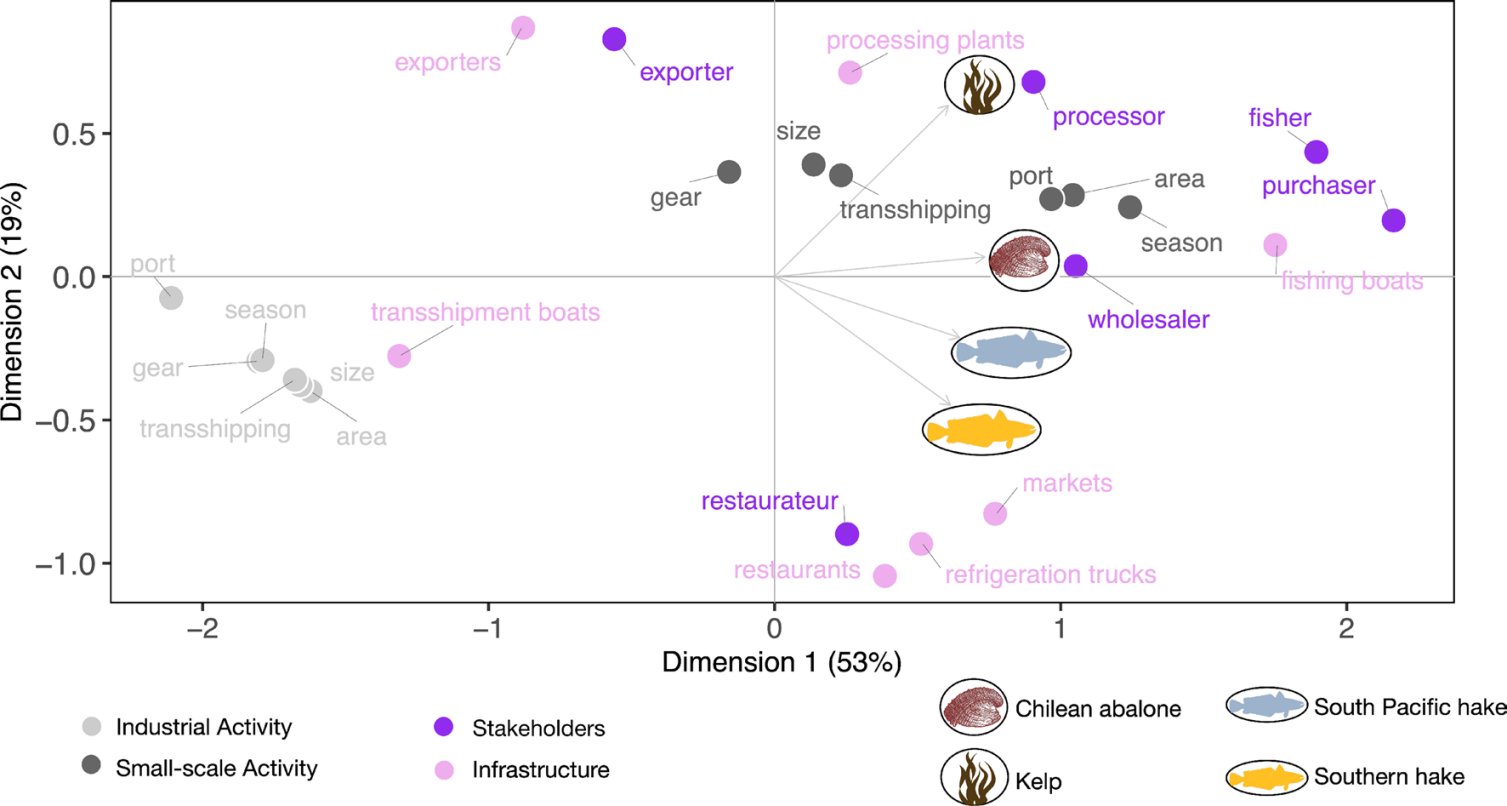
Principal Component Analysis (PCA) biplot of four focal fisheries and factors connected to illegality. The PCA score plot (colored circles) represents how the levels of each factor cluster (or not) across the two principal components, while the loading plot represents how each fishery is influenced by each principal component. Represented on the horizontal dimension, all the fisheries share the same high levels of small-scale activity (i.e., port, quota, and season) and stakeholders (i.e., fishers and purchasers). Represented on the vertical dimension, differences between the focal fisheries are revealed in the infrastructure and certain stakeholders. Factors connected to exportation and processing are closely connected to kelp illegality, while factors connected to retail (e.g., restaurants and markets) are closely connected to hake illegality. For all fisheries, industrial activities and transshipment boats are weakly associated with illegal activity.

The PCA analysis of the model results from the fishery profiles provides insights into the Chilean IUU landscape. The first two dimensions explained 72% of variability in the data, with the first dimension explaining > 53% (Fig. 3). All four focal fisheries were strongly positively correlated with the first dimension (r > 0.68; Table S4), meaning that they all had high scores for the factors on the right, intermediate scores for factors close to the center of the coordinates and low scores for factors on the left (Fig. 3). All four fisheries contributed to the first dimension, with Chilean abalone contributing the most (Fig. S6). Stakeholders and infrastructure with the highest scores for the four fisheries included fishers, purchasers, and fishing boats. All industrial activities had low scores for the four fisheries, while illegal small-scale activities related to landing port, quota, and season had high scores. Kelp and southern hake contributed the most to the second dimension (Fig. S6), with the former positively correlated (r = 0.7) and the latter negatively correlated (r = − 0.50; Table S4). Kelp is associated with higher estimates for exporters and export vehicles, along with processors and processing plants. In contrast, southern hake is associated with higher estimates for restaurateurs, restaurants, markets, and refrigeration trucks (Fig. 3).

## Discussion

As a premier threat to effective fisheries management and conservation^5^, barriers to addressing IUU fishing include practical, repeatable, and affordable ways to monitor and prioritize investments to address illegal activity. By working with government fisheries enforcement officers, we were able to rank and estimate illegal activity at a national scale. Our approach provides an opportunity for fisheries agencies to formalize their institutional knowledge, while accounting for potential biases, such as experience and differences among individual officers. The use of online surveys and statistical models provides a framework which agencies can use to monitor illegal fishing activity, discover leverage points to target investments in enforcement operations, and evaluate the impact of their interventions.

We estimated relative illegal activity for 20 Chilean fisheries, which make up ~ 2 mm metric tons and accounts for ~ 70% of wild capture landings in 2018^33^. Our results provide evidence of significant illegal activity in the small-scale fishing sector, highlighting the need to recognize and address the specific impacts of illegality on small-scale fisheries as a key component of designing solutions to reduce IUU fishing. Small-scale fisheries and its unique characteristics are often under-appreciated from IUU fishing assessments and interventions^42^. For example, new regulations to tackle IUU fishing in Chile have focused on increasing prosecution and strengthening sanctions (e.g., imprisonment), irrespective of the sector or fisheries. This approach implicitly frames IUU fishing through lenses of industrial fishing and organized crime, raising the risk of disregarding the diversity and legitimacy associated to other approaches to addressing illegal fishing^42^. Addressing IUU fishing within small-scale fisheries must consider the legitimacy of measures^43^. This necessarily implies tailoring solutions with a small-scale fishery perspective, such as strengthening management rules and compliance^24^.

Our results are broadly consistent with previous research that presented evidence of high levels of illegal fishing in three fisheries: south Pacific hake, Chilean abalone, and southern king crab^15,24,44–48^. Working directly with fishers, for example, researchers empirically estimated illegal catch of Chilean abalone to be between 70 and 86% of the total harvest in central Chile^15^. Chilean abalone had the second highest ranking, after controlling for experience and 33% and 31% of respondents estimated national illegal landings to be between 20–50% and 50–100%, respectively (Fig. S1). Similarly, researchers estimated the total catch of south Pacific hake to be two times the official landings^48^. The fishery had the highest illegal estimate in our model, and 39% and 42% of respondents estimated national illegal landings to be between 20–50% and 50–100%, respectively. The same researchers estimated illegal southern hake landings to be ~ 75%^48^, while it was the third highest estimate in our model and 59% of respondents estimated national illegal landings to be between 20 and 50%. For both hake species, previous studies estimated that illegal landings were significantly greater for the small-scale sector compared to the industrial sector^48^. Further research on integrating expert elicitation and estimation methods could prove useful for improving IUU estimations.

Our results also produced new information on illegal activity in Chile, including relative high estimates for red-cusk eel (*Genypterus chilensis*), southern rays bream (*Brama australis*), and anchoveta (*Engraulis ringens*), of which the latter fishery accounted for ~ 40% of annual landings in 2018^33^. The fishery profiles provide additional layers of information on stakeholders, infrastructure, and activities within sectors. For Chilean abalone, for example, illegal activity appears to be dominated by small-scale fishers (fishing boats), restaurateurs (restaurants), and buyers (markets). In contrast, illegal activity in the kelp fishery is dominated by stakeholders and infrastructure connected to processing and exporting. While IUU activities are often dynamic^49^, fisheries profiles can help identify or confirm factors that may not be important in the illegal activity of a specific fishery, and thus resources can be prioritized elsewhere (e.g., illegal landings for southern and South Pacific hake).

The multivariate analysis provides a relatively simple tool to easily visualize factors that are similar and different across multiple fisheries of interest. Certain stakeholders (i.e., fishers and purchasers) are viewed as important for illegal activity for all four fisheries. Yet, different stakeholders and infrastructure are considered important with respect to illegality associated to kelp and hake. Factors associated to exporting and processing are important for kelp, while domestic factors are more important for hakes, such as markets and retail activity. The ability to use surveys to create multi-factorial fishery profiles can inform the design of specific interventions to reduce illegality, while the multivariate analysis can inform the broader IUU landscape, including the design of interventions that could have synergistic impacts on multiple fisheries.

Researchers and practitioners have highlighted the need of improved frameworks and knowledge systems to address complex problems such as illegal fishing^50^. Relatedly, methods to measure and monitor the illegal use of natural resources should strive to be cost effective, time efficient, and statistically rigorous^51^. While the use of expert judgement to inform estimates of illegal fishing is not uncommon, it is often informal and unsystematic^11^. Our expert elicitation approach, using online surveys, provides a fast and cheap means of systematically formalizing expert judgement, while also controlling for potential biases. Thus, it is likely to be highly complementary to other approaches to characterizing IUU fishing. The approach has a number of advantages. First, while the respondents remain confidential, there is transparency and consistency with the pool of experts: all are fisheries enforcement officers identified by the SERNAPESCA Director for their knowledge and experience on IUU fisheries. Second, our fisheries-level estimates do not rely on problematic sample sizes (n > 30), which complements other approaches that rely heavily on just a few confidential sources^18,21^. Third, we statistically adjust for respondent and his or her expertise, which allows for the identification and control of potential biases. Last and perhaps most important for impact, it is scalable: almost all countries have fisheries enforcement officers and we implemented the survey with SERNAPESCA over a matter of months. Any expert elicitation process, of course, has its limitations. While the methodology enjoys strong support in the literature, experts can obviously be subject to biases^52^. Thus, mitigating for any potential biases through careful sampling designs and statistical methodologies are important, as well as attempts to complement and triangulate the approach with other methods and data when possible (e.g., IUU infractions).

Eliciting information from fisheries officers provides insights on how government institutions can build a knowledge base on IUU fishing activity, which can be used to shape the broader institutional context in which they operate. While expert opinion approaches do not replace empirical research, our Bayesian modeling provides a statistically rigorous methodology that improves parameter estimates, while also controlling for potential biases^27^. This is particularly important for monitoring activity through time with different enforcement officers. Our approach can also be expanded (e.g., additional surveys) to estimate related phenomena suspected of influencing illegal activity in complex ways (e.g., diversion capacity). By estimating illegal activity across activities, stakeholders, and infrastructure, our approach can help address fragmentation of knowledge that is suspected of preventing effective enforcement^51^. A combination of methodologies and approaches will always be preferred to measure and monitor illegal and clandestine behavior^51^. Expert elicitation from fisheries officers is likely to be widely embraced by fisheries agencies, as evidenced by SERNAPESCA's interest in institutionalizing the tool (Alicia Gallardo, Director of SERNAPESCA, personal communication). Given that current approaches to measuring IUU activity can be resource intensive and sometimes controversial^19,21,53^, estimating illegal activity directly from fisheries enforcement officers is a complementary approach that provides a cost-effective, rapid, and rigorous method to measure, monitor, and inform solutions to reduce IUU fishing.

## Supplementary information

Supplementary information

## Data Availability

The datasets generated from the current study are available from the corresponding author on reasonable request.
