# Author Correction: Estimating illegal fishing from enforcement officers

**DOI:** 10.1038/s41598-021-95745-6

**Published:** 2021-08-10

**Authors:** C. Josh Donlan, Chris Wilcox, Gloria M. Luque, Stefan Gelcich

**Affiliations:** 1 Advanced Conservation Strategies, Midway, UT 84049 USA; 2 Cornell Lab of Ornithology, Cornell University, Ithaca, NY 14850 USA; 3 CSIRO Oceans and Atmosphere, Castray Esplanade, Hobart, TAS Australia; 4 Center of Applied Ecology and Sustainability (CAPES) & Center for the Study of Multiple‑Drivers on Marine Socio‑Ecological Systems (MUSELS), Pontificia Universidad Católica de Chile, Santiago, Chile

Correction to: *Scientific Reports* https://doi.org/10.1038/s41598-020-69311-5, published online 27 July 2020

The original version of this Article contained a repeated error in the Methods section, under the subheading ‘Experimental design’, in the Results section, under the subheadings ‘Fishery profiles’ and ‘Principal component analysis’, in Figure 2, in the caption of Figure 3, and in Figures S2-S5 of the Supplementary Information file, where

“area”

now reads:

“quota”

The original Figure 2 and accompanying legend and the original Supplementary Information file are provided below. The original Article and accompanying Supplementary Information file have been corrected.

**Figure 2:**
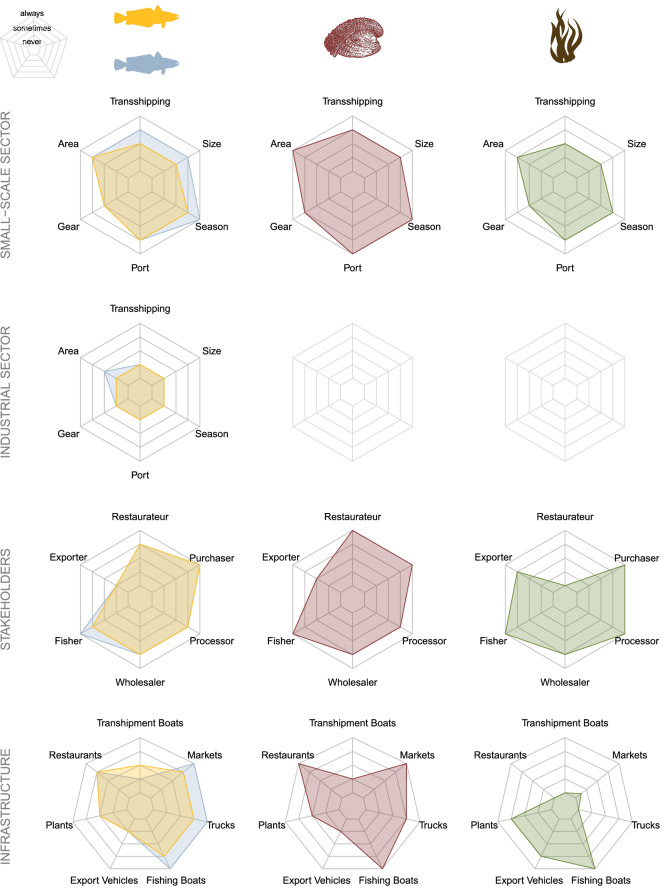
Fishery profiles of four Chilean fisheries: south Pacific hake (blue), southern hake (orange), Chilean abalone (red), and kelp (green). Predicted median estimates of the level of illegality in industrial sector activity, small-scale sector activity, stakeholders, and infrastructure. Industrial fishing does not exist for Chilean abalone and kelp. Predicted medians are from a Bayesian cumulative multinomial logit model for each of the four focal fisheries. The entire posterior distributions of the model results are shown in Figs. S2–S5.

## Supplementary Information

Supplementary Information.

