# Supplementary material for: Estimating illegal fishing from enforcement officers
Source: Sci Rep. 2020 Jul 27;10:12478. doi: 10.1038/s41598-020-69311-5 (PMC7385102; doi:10.1038/s41598-020-69311-5)
Supplement: Supplementary file 1 — Supplementary information [file 41598_2020_69311_MOESM1_ESM.pdf]

Supplementary Materials
Estimating Illegal Fishing from Enforcement Officers

C. J. Donlan[1,2*], C. Wilcox[3], G. M. Luque[1], S. Gelcich[4,*]

[1] Advanced Conservation Strategies, Midway, UT, 84049, USA
[2] Cornell Lab of Ornithology, Cornell University, Ithaca, NY, 14850, USA
[3] CSIRO Oceans and Atmosphere, Castray Esplanade, Hobart, Tasmania, Australia
[4] Center of Applied Ecology and Sustainability (CAPES), Pontificia Universidad Católica de Chile, Santiago, Chile

[*]Corresponding authors: jdonlan@advancedconservation.org; sgelcich@bio.puc.cl

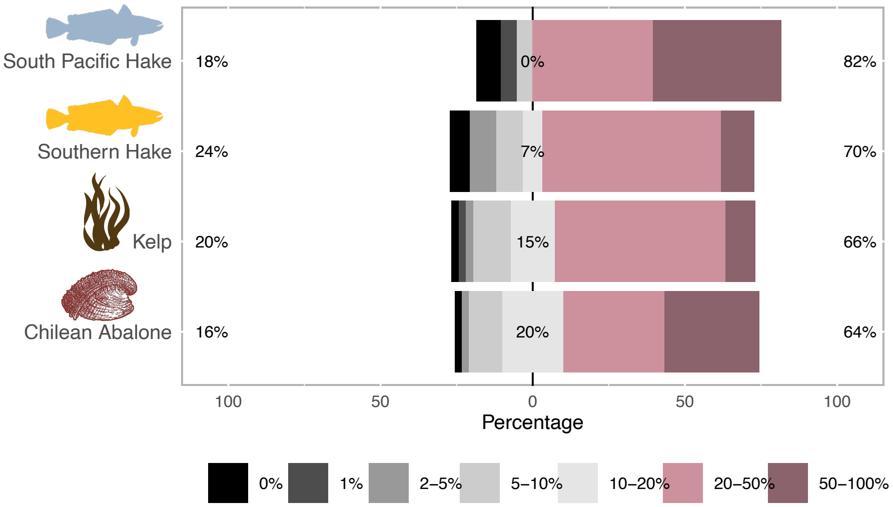

Figure S1. Percentage of total landings coming from illegal activities as reported by Chilean fishery enforcement officers. Distribution of seven possible responses for four focal fisheries.

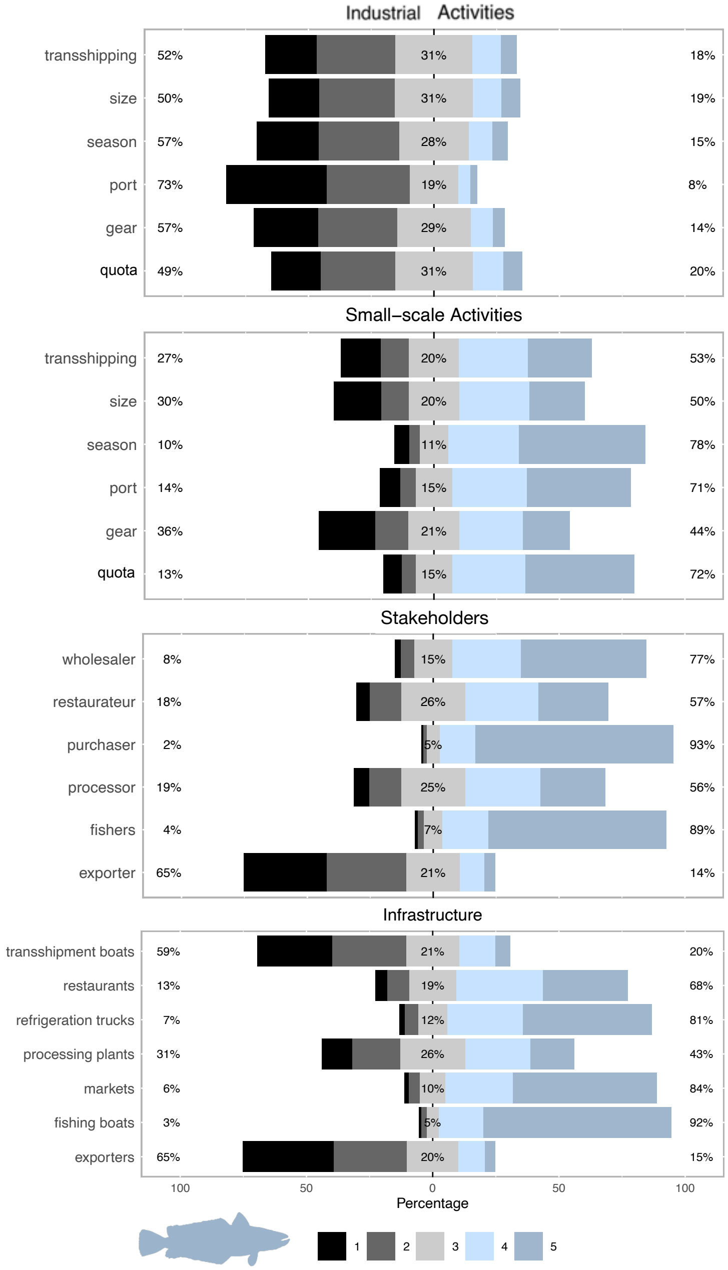

Figure S2. Distribution of predicted estimates for south Pacific hake (*Merluccius gayi gayi*) for level of illegality for industrial sector activity, small-scale sector activity, stakeholders, and infrastructure. From a Bayesian cumulative multinomial logit model that controls for respondent and experience.

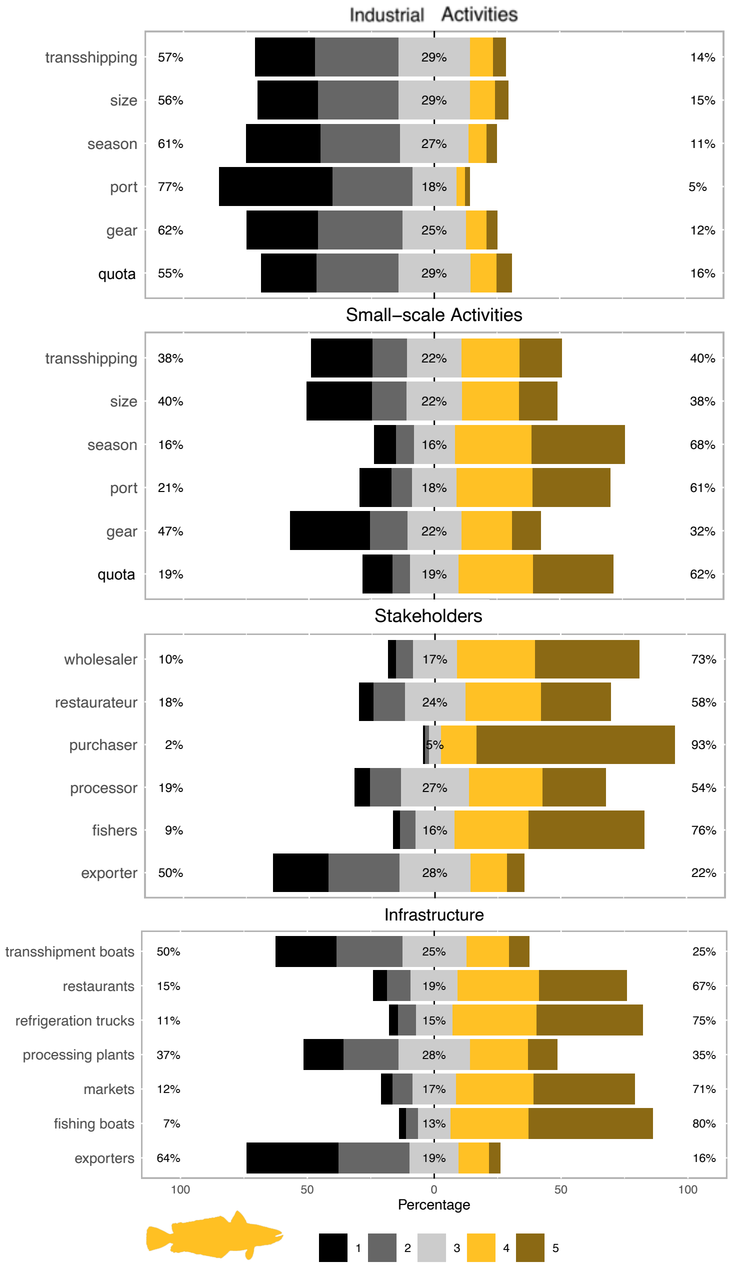

Figure S3. Distribution of predicted estimates for southern hake (*Merluccius australis*) for level of illegality for industrial sector activity, small-scale sector activity, stakeholders, and infrastructure. From a Bayesian cumulative multinomial logit model that controls for respondent and experience.

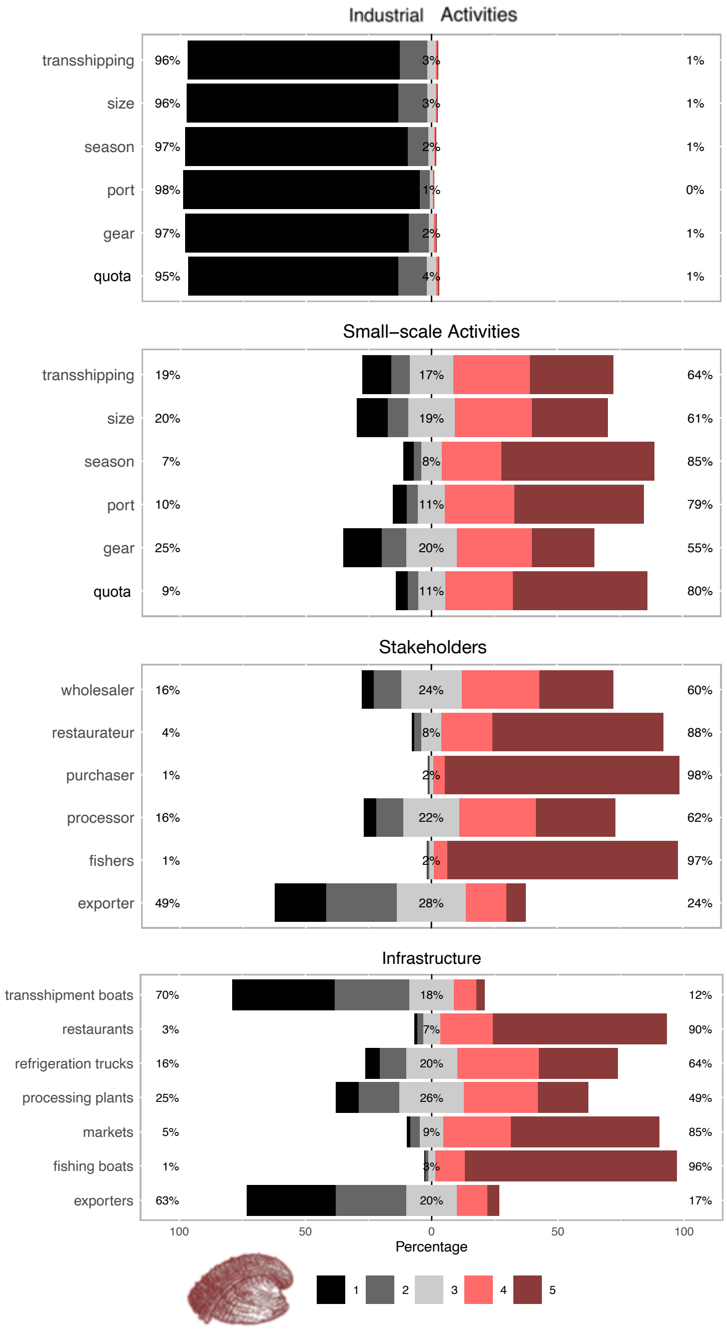

Figure S4. Distribution of predicted estimates for Chilean abalone (*Concholepas concholepas*) for level of illegality for industrial sector activity, small-scale sector activity, stakeholders, and infrastructure. From a Bayesian cumulative multinomial logit model that controls for respondent and experience.

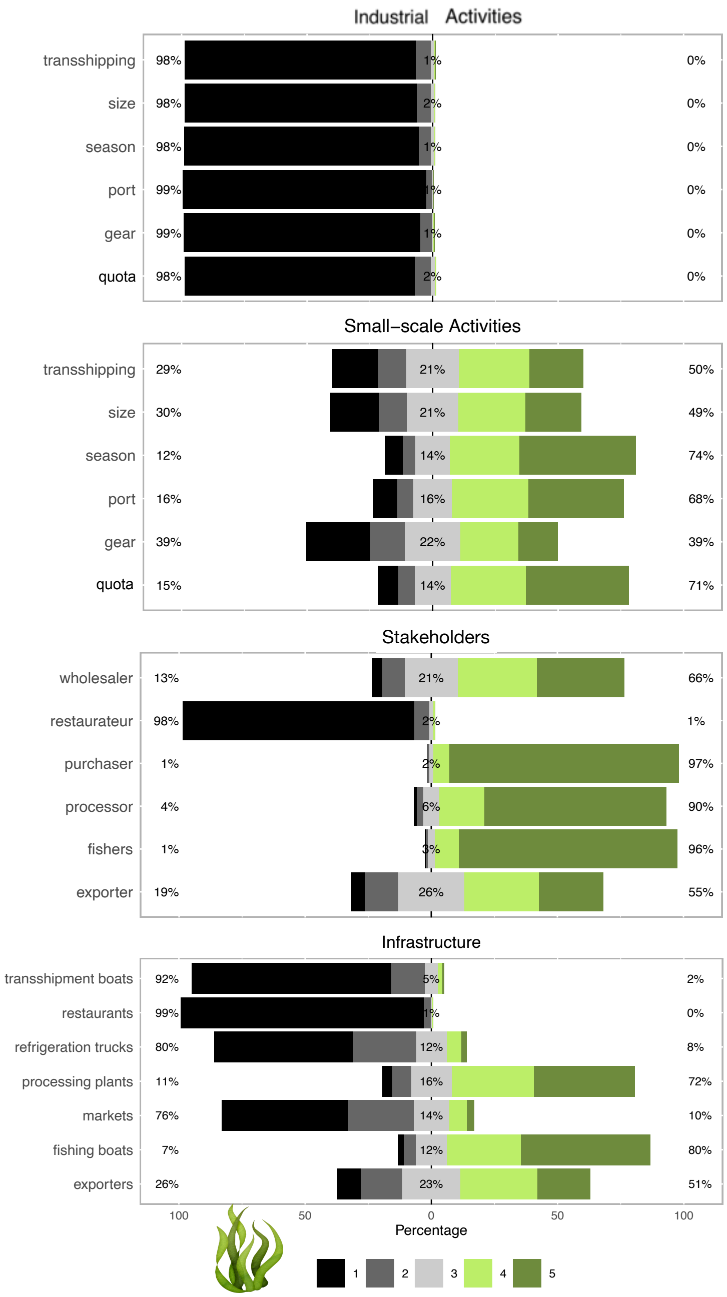

Figure S4. Distribution of predicted estimates for kelp (*Lessonia spicate, L. berteroana, L. traberculata, Macrocystis pyrifera*) for level of illegality of industrial sector activity, small-scale sector activity, stakeholders, and infrastructure. From a Bayesian cumulative multinomial logit model that controls for respondent and experience.

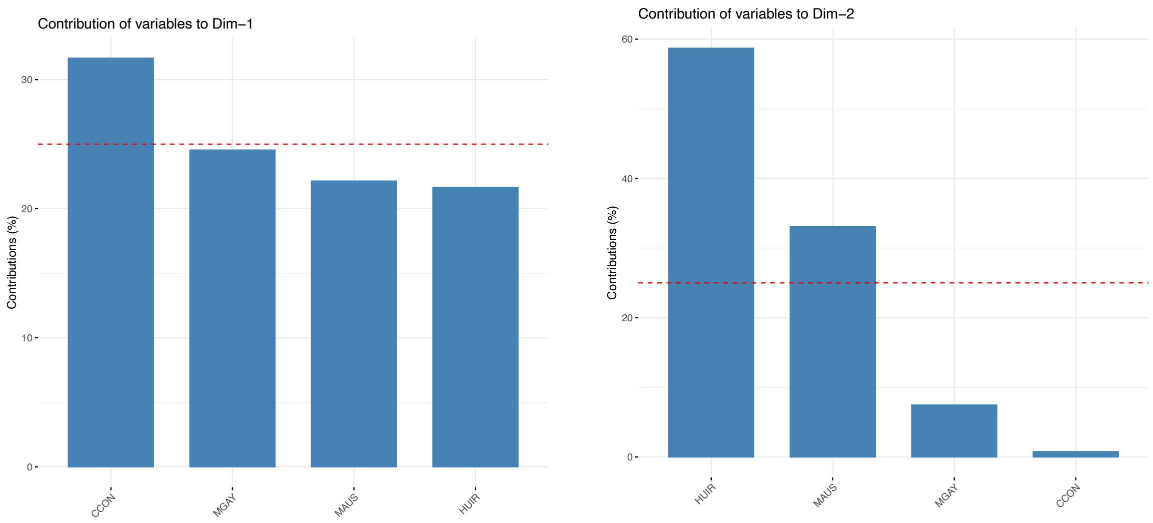

Figure S6. Contribution of variables (i.e., fisheries) to the two dimensions of the Principal Component Analysis (see Fig. 3). The four fisheries included are Chilean abalone (CCON), south Pacific hake (MGAY), southern hake (MAU), and kelp (HUIR).

Table S1. Model performance assessed by *leave one out cross validation*. Lower cross validation scores indicate better predictive ability. Positive differences between models indicate an improvement in the second model. Standard errors were approximately normally distributed; thus, the error term +/- 1.96 gives the upper and lower 95% confidence intervals on the scores. The two models are significantly different at this level.

**Model**	**Cross validation score**	**Standard Error**
Null model: Respondent (random)	2734.73	36.09
Model 1: Illegal Score (fixed) + Respondent (random)	2465.52	42.64
Null model - Model 1	269.21	31.97

Table S2. Parameters for a linear model of the effect of experience on the difference between fisheries scores by respondents and the values predicted by a model across all respondents.

**Parameter**	**Estimate**	**Standard Error**	**p value**
Intercept	0.52	0.07	<0.001
Experience Score	-0.16	0.02	<0.001

Table S3. Differences among models for nominal illegality scores, comparing alternative structures for incorporating respondent experience. Lower cross validation scores indicate better predictive ability. Positive differences between models indicate an improvement in the second model. Standard errors are approximately normally distributed; thus, the error term +/- 1.96 gives the upper and lower 95% confidence intervals on the scores.

Model	Cross validation score	Standard Error
M1: Illegal $score_{(fixed)}$ + $respondent_{(random)}$ + experience	2226.64	48.26
M2: Illegal $score_{(fixed)}$ + $respondent_{(random)}$ + $experience_{(categorical)}$	2225.45	48.27
M3: Illegal $score_{(fixed)}$ + $respondent_{(random)}$ + $experience_{(linear)}$	2225.48	47.93
M4: Illegal $score_{(fixed)}$ + $respondent_{(random)}$ + $experience_{(smoothed)}$	2224.70	48.15
M1 - M2	1.19	0.43
M1 - M3	1.15	5.30
M1 - M4	1.94	1.91
M2 - M3	-0.03	5.31
M2 - M4	0.76	1.93
M3 - M4	0.79	3.70

Table S4. Correlation matrix between the four quantitative variables (i.e., fisheries) and the two principal component dimensions.

Dimension	Fishery	Correlation	p value
1	Chilean abalone	0.82	<0.001
1	South Pacific hake	0.72	<0.001
1	Southern hake	0.68	<0.001
1	Kelp	0.68	<0.001
2	Chilean abalone	0.66	<0.001
2	South Pacific hake	0.08	<0.001
2	Southern hake	0.24	<0.001
2	Kelp	0.50	<0.001
